# Perceptions of the usefulness of an online simulated clinical examination

**DOI:** 10.5116/ijme.679e.067d

**Published:** 2025-02-27

**Authors:** Arunaz Kumar, Mahbub Sarkar, Paul Fullerton, Jodie Vickers, Peter Barton

**Affiliations:** 1 Faculty of Medicine, Nursing & Health Sciences, Monash University, Melbourne, Australia

**Keywords:** Observed Structured Clinical Examination OSCE, Online assessment, Clinical assessment, Online examination

## Abstract

**Objectives:**

This study aims at evaluating the role of Monash Online Simulated Clinical Examination (MONSCE, where students demonstrate their clinical consultation, problem solving and counselling skills in a virtual encounter) in relation to the Observed Structured Clinical Examination (OSCE). The study addresses feasibility and application, student, tutor and Simulated Patient (SP) acceptance and also assessing future role in student assessment.

**Methods:**

Drawing on social constructivism, the study employed a qualitative methodology to explore perspectives of medical students, examiners and SPs across metropolitan Melbourne, rural Victoria and Malaysia. Data included individual interviews with nine examiners, eleven SPs, and three focus groups with students. Data were transcribed and thematically analysed using framework analysis.

**Results:**

Analysis demonstrated overlapping perspectives with five themes - fit for purpose assessment, focus on dynamics of online discourse, perceiving realism, readiness for practice and implications for future, with ongoing role in Telehealth. Readiness or preparation for practice was acknowledged through impact on student performance for progression, examiners’ focus on assessment rigour replicating chaos and complexity of real life and SPs drew analogy with real-life clinical consultations.

**Conclusions:**

MONSCE assessments appear to be useful for student assessment of skills like history taking and clinical counselling. Their role was considered complementary to in-person clinical skills assessment but not replace the complexity of real life or replicate skills assessment of empathy, physical examination, and difficult communication, where in-person assessment may be preferred.

## Introduction

Assessment in the field of medical education has been constantly evolving over decades, mostly utilising a mix of written and clinical simulated patient assessments.  The Objective Structured Clinical Examination (OSCE) was introduced in 1975.^1^^,^^2^  It standardised (simulated) patient interaction and reduced the effect on student scores due to variability in patient interaction. It created an effective, valid instrument for assessing cognitive, affective and psychomotor skills that could be applied to a battery of clinical settings for both formative and summative purposes.^3^

Over the last few years, assessments have evolved to suit the changing landscape of teaching and learning. The Covid-19 pandemic triggered replacement of in person teaching with online learning and curriculum delivery, inclusion of online consultations (Telehealth), where students participated by joining the online clinical discourse.^4^ Similarly, students have been encouraged to be a part of virtual handovers and ward rounds to facilitate student participation in clinical work-based activities, with virtual participation. The conversion of in-person clinical interactions has not just been limited to learning, but also in online summative assessment^5^^, ^^6^ with many initiatives retained, to continue post-pandemic. However, these online assessments may not have been evaluated, especially in relation to what they may achieve/ seek to achieve in comparison to the OSCE. Our study aims to fill the gap of comparing online clinical assessments with OSCE.

Hence, we introduced Monash Online Simulated Clinical Encounter (MONSCE) for end of the year summative exam, that involves a student, SP, and an examiner present together in a virtual clinical encounter. The students were presented with a clinical scenario, requiring them to immerse themselves in the clinical consultation with the patient, either to obtain a history and examination findings (such as in the case of psychiatry or general practice assessment) to reach a diagnosis, or use available clinical information to advise treatment and counsel the patient (such as explanation of surgical procedures or medications); sometimes they did both. Through the timed interaction, with pre-prepared SP prompts (like OSCE), the students had an opportunity to demonstrate their clinical consultation, problem solving and counselling skills.  On most occasions, the examiner would be a silent (invisible online) observer of the student-SP interaction, responding to students only when examination findings or investigation results were requested.  Otherwise, the student-SP interaction was like an online doctor-patient consult.

Benefits and ease of administering the MONSCE were considered; with a more standardized approach, the session could be recorded online with ease and viewed later by other examiners to ensure that examiners were consistent in their marking approach.  Hence the replacement of MONSCE in place of OSCE was proposed with the potential to improve inter-rater reliability and maintain a standardized approach to marking all students participating in the exam. Co-location concerns were another reason affecting feasibility of OSCEs (to simulated patients, students, and examiners). The ability to examine remotely meant that the location of each actor was independent: they didn't need (unlike OSCEs) to be co-located. Monash University has a total of nine campus locations (three of which are rurally located in the state of Victoria and an international campus in Malaysia). This enabled international examination (Malaysia and Victoria were mixed for major cohorts), with convenience of not travelling or large-scale venue booking required for OSCE. It was considered that if MONSCEs were found to be sustainable, valid, and easily applicable, they may continue to have a role in future ongoing assessment.

Hence, a broad-based qualitative evaluation involving all stakeholders was undertaken to explore comparison of MONSCE with the pre-existing OSCE. The research questions for the study were:

    1.      What were student, examiner and Simulated Patient (SP), perspectives on online and in-person clinical skills assessment and what did these online (MONSCE) and in-person assessments (OSCE) achieve?

    2.      What was the impact of MONSCE (in comparison to the previously administered OSCE) assessments on readiness for future clinical practice?

## Methods

### Study design

The study followed a qualitative research design with evaluation of MONSCE and OSCE with triangulation from all key stakeholders involved in or impacted by the assessment process – the students who attempted the MONSCE (and OSCE in the preceding years), the examiners with experience in examining both OSCE and MONSCE, and simulated patients who have had experience of the OSCE in the past and were posing to be patients  for the MONSCE assessment, at the time the study was  conducted. The study was designed to inform the assessment approach on how it should be retained or modified further in future years. Hence the evaluation of all stakeholders involved or effected by the assessment, was considered as it would guide and develop future assessment initiatives.

#### Theoretical underpinning

We employed a social constructivist viewpoint where knowledge is created and applied in a socially mediated context by individuals; in this case the knowledge is the understanding of MONSCE and OSCE assessment process (and what it achieves/seeks to achieve) and the individuals contributing to creating that knowledge are students, examiners, and the Simulated Patients (SPs). As described by Vygotsky ^7^, the learner is an active participant in the learning process, and knowledge results from the learner’s interaction with the environment; the content of learning is closely aligned with the process of acquiring that learning.  Social constructivism also embraces that the tension resulting from individuals with conflicting thoughts, contributes to learning and is the very stimulus for creating new knowledge. And lastly the social environment plays a key role in creating that knowledge, even when the environment may pose to challenge the belief, it still assists in contributing to existing knowledge, hence, leading to new knowledge. The study was conducted from January 2020 to December 2022.

#### Participant recruitment

Monash Medicine has nine campuses including metro, rural and Malaysian campus for all clinical year levels. The summative MONSCE exam replaced the previously conducted onsite OSCE for all sites, and Year level 3 and 4 (clinical years for medical students with Year 5 being the pre-intern year with no formal summative end of the year exam). A combination of purposive and convenience-based sampling of student, examiner and simulated patients was considered, to ensure participation from all locations and from both year 3 and 4 students and examiners. For ethical consideration, the evaluation was considered to not cause anything more than minor discomfort to the participants, in reflecting upon their examination experience; hence, a low-risk approval was obtained from Monash University Human Research Ethics Review Committee (MUHREC – 30460). If any student was to experience distress by reflecting on the examination process, measures were established to ensure adequate support and counselling.

#### Students

The project was publicized by online medical student forums and other student social groups through the newsletter. On completion of the End of the Year (EOY) exams, medical students from all sites attempting the MONSCE (in Year 3 and 4 of the 5-year medical degree course and had participated in the OSCE in year 1 and 2), were invited to attend focus groups to capture their thoughts. It was considered that student participants may have had a variable experience from different locations -  hence metropolitan, rural and Malaysian students were recruited., but allocated to mixed heterogenous focus groups to capture their individual experiences through discussions.

#### Examiners

Examiners (from varying professional characteristics and sites), who had examined both OSCE and MONSCE, were offered an online semi-structured interview for 30-40 minutes to get deep insights into the challenges faced by them in designing and implementing the new assessment. Examiners were invited by the administrative lead at the university who was also on the research team, JV, (instead of an academic lead to avoid any differences in power and minimising participant perception of coercion). They were reassured that their responses would not affect their employment or teaching role.

#### Simulated patients

Simulated patients (SPs) of different age groups and gender, and had participated in both OSCE and MONSCE, were offered an interview lasting 30-40 minutes, exploring their experience of participating in the MONSCE.

### Data collection

Qualitative data were collected through student online focus groups, individual examiner, and Simulated Patient (SP) interviews. Focus groups were chosen for data collection from students, as there may be a wider variation in learning based on individual student experiences, and geographical location of their clinical placement (in metro areas versus rural placements) and to create a safe space for students to communicate without inhibition, and gain collective insights about their assessment.^8^ Hence mixed heterogenous focus group with students from various locations and both year levels were conducted online and recorded (using Zoom meetings). Similarly, individual examiner and SP interviews were conducted online to gain deep insights regarding their experience.

A total of three student focus groups with 6-8 participants in each group was conducted. Nine examiner and eleven SP interviews were conducted ensuring participation from metro, rural and Malaysian campus. The research questions were thought to have a narrow focus; hence, these numbers were considered sufficient to address it with adequate information power to answer the research question, which was also confirmed after data analysis. Student participants were reassured that their participation (or not) and their responses would not affect their exam results and progression decisions, through an explanatory statement and a consent form, which was also used for the examiners and SPs. Participants were reassured of confidentiality and that the anonymous pooled research data would be shared in the form of conference presentations and publications. Examiners were asked about their experience of participating in the MONSCE with questions like “How are the MONSCE assessing students’ history taking skills or understanding of clinical management compared to OSCE?” They were also asked about their observations on impact of MONSCE on student performance and readiness for the next level as a pre-intern year. Questions to the SPs, specially probed into “How did the students communicate?” and “Did they express empathy and understanding of the SPs problems, did students offer satisfactory responses (or not), where relevant?”. All interviews and focus groups were audio-recorded and transcribed verbatim.

#### Research team and reflexivity statement

The study was designed collaboratively by the research team, which consisted of lead researcher AK (academic clinician with an evaluation role) PB (academic clinician and chief examiner), MS (academic qualitative researcher), PF (academic clinician lead for the Malaysian campus) and JV (administrative lead for the MD program). To avoid any risk of coercion, all student focus groups were conducted by the lead researcher AK, who is independent of the assessment process.  All SP and examiner interviews were conducted by AK, and PF, AK was not known to students and SPs, but known to some examiners. PBs role was in study design and analysis of transcripts and writing.  He did not participate in data collection or listening to audio files. To prevent coercion, JV approached the examiners and students through online communication with an offer of voluntary participation. All members of the research team maintained a diary to identify and share their personal bias and reflections at the start and continued at various points during the study.

### Data analysis

The transcripts and their recordings were shared with the research team to confirm accuracy. AK analysed all transcripts while all others were shared by the team with each transcript reviewed independently and inductively by two to three researchers, (PB, PF and MS) and later shared with JV prior to reaching final consensus. The transcription software *Otter AI *was used and edited further by AK after listening to recordings*. *AK checked all transcripts for accuracy prior to sharing the transcripts, although the audio-recordings were also shared with the research team (except PB) for verbal nuances to assist in accuracy in data interpretation. Only anonymous version of transcripts was shared with PB. After initial familiarisation with the data  and  frequently reverting to the recordings (to identify nuances), a coding framework was developed using guidelines described by Ritchie and Spencer,^9^ for thematic analysis. A detailed index of the coding data was created independently by all researchers group consensus further allowed to jointly develop and refine the coding through subsequent meetings after the initial round of analysis. Through multiple rounds of analysis and mapping the data, the final themes were developed with grouped subthemes, agreed upon.

## Results

Analysis demonstrated overlapping perspectives with common themes in the study groups; the most prominent theme related to the dynamics of discourse comparing online with in-person interaction, especially in the context of rapport and connection built between the student and the SP, followed by motive and value of the exams: assessing fit for purpose for history taking and counselling; inadequate for physical exams/procedural skills or complex counselling like “breaking bad news”. The third theme was on perceiving realism where participants compared the in-person interaction to a ward round and even to a real patient clinical assessment. They referred to how subtle nuances can affect clinical diagnosis and management, and the realism of the exam environment, where being on the “screen” was less stressful than in a physical exam setting.  The final two themes were on readiness of future practice and exam implications for the future (role in Telehealth).

### 1) Dynamics of Discourse – (Communicating differently online versus in-person)

This theme was based on discussion on how the online interaction differed from in-person. Most SP participants suggested that “eye contact” was missing as students were looking at the camera and they were sometimes not able to read the SPs body language. However, it was convenient and didn’t seem to impact student performance. Both examiners and students themselves described facing the challenge of missing out on subtle cues of facial expression.

“But I guess that in person connection is, kind of lost. And I think it is a bit harder to read into, you know, the simulated patient when it is online, I think that's the only sort of barrier and, and I guess, disadvantage with it being run online now. But apart from that, I think it's yeah, it's definitely saved a lot of time in efficiencies.” (SP-B, Male)

“So. all good students perform well on it, and the not so great, would still be struggling the same way… you still see those ABCD students, they still come across whether it's zoom or in person, but then it's a personal thing, because you find a lot of people want a need to have the the face to face interaction. And that's really important to them, whereas others are indifferent. So yeah, it's a very personal thing, I think.” (SP-R Male)

“I think there's probably pros and cons of both ways. I think the benefits for face to face for me is, Just I think it's a bit more personal. And I think even I don't know, my perception would be the candidates, it might be a little bit less stressful if, you're actually dealing with a human being versus you've got this examiner, that's just a blank screen on Zoom, I think that potentially might add to the stress for the students.” (Examiner-S, Male)

“At the end of the day, when we're practicing medicine, it's not online, it's very much in person doing Doctor things. And I think having examinations online as a whole, it's not the greatest in terms of building, you know, our communication skills and people skills.” (Student FG2)

### 2) Fit for purpose (Comparing suitability of online and in-person based on skills being tested)

This theme indicated differences where MONSCE was suitable (or not) compared to the OSCE. All three participant groups suggested that MONSCE was appropriate for tasks like taking a history from the patient, giving simple advice, offering treatment options, and discussing surgical procedures, or seeking consent. However, tasks where any physical examination skill needed to be assessed were not possible and neither was offering complex counselling e.g. alcohol cessation or addressing social complexities such as domestic violence. All three participant groups conveyed similar perspectives and this theme was found to be the key focus of discussion in debating merits of MONSCE and OSCE.

“I think I think in certain types of cases, there's not a lot of difference in doing a face to face or MONSCEs like history taking stations or explanation, sort of stations, there is obviously a difference in, in communication with the candidate in the way they communicate, perhaps and the way they present themselves”. (SP-P Female)

“…if you're trying to encourage them to cut down on drinking, or console them because their child's just been diagnosed with something? Well, you know, in real life, it's, it's going to be much better one on one in person.” (SP-D Female)

“ ..if the exams are purely to see if they can have a basic conversation take a history and diagnose based on the information they've been given that I don't think it matters, but… if the exam is partially to put them under actually a little bit of pressure, and, and have them in a room where they're being watched? And they have to step up and like really have their have to hold themselves together… which is see how they react under stress.  Then you have to do a one on one” (in person). (SP-T Male)

“History taking, let's say, interpretation of lab results, might be discussing or explaining a diagnosis to a patient, the sort of things or explaining the management plan to the patient, all those sort of skills would be obviously completely suitable [for the MONSCE], In OSCE I think like breaking of bad news and showing empathy”. (EX-S, Male)

“I think exam wise, me personally, if I can rattle off an exam easy, but like, for instance, today, I percussed the patient's chest like yeah, you can say I would now percuss the patient's chest like doing it like I can't hear anything like my percussion is absolutely terrible. But like in an MONSCE I would have been fine because all I would have had to say is ‘I would then percuss the chest’ and like I know what to interpret, resonant and dull but I can't hear that when I do it in actual life.” (Student - FG3)

### 3) Perceiving realism (Comparing assessment to a doctor-patient interaction)

Examiners and SPs compared student interactions with SPs to a doctor-patient consultation. All three groups commented on the examination environment with online waiting rooms and moving physically from room to room replicating the chaos of real clinical work life but could be a distraction from the exam itself. Students also discussed the stress felt while in the physical setting of an exam hall as opposed to being in the comfort of their rooms.

“…it's how close we stand or sit to each other what we do with our bodies, at the moment on Zoom, I can move around my hands, literally, you're only seeing my very top half. But when you're in person your whole every, the way you're sitting, what you do with the legs, how far apart you are. The eye contact is, is a bit different for for zoom, it's it's just got that fake element, if I can, how do you say it's a sense of unreality?” (EX-K, Female)

“…the online environment is kind of important. You know, you need to get the camera well set up and good lighting and you know, you need to make sure the background doesn't have, you know, bottles of alcohol or dogs or pets or other things that might be distracting to them, because I suppose it's respectful to the patient.” (EX-S Male)

“Because it's different when you're with a human, your endorphins, everything is different. You just feel different when you're with a human. But then, again, some students may prefer the MONSCEs, because they may feel that it gives them some sort of comfort that they're not in the room...” (SP-R, Male)

“I feel like OSCEs had that sense of formality and had a rigorous process and requested for certain amount of time that was stamped on doors or bells, who are people monitoring us”.  (Student FG1)

### 4) Readiness for practice (Comparing authenticity of assessment to clinical practice)

“Readiness for future practice” demonstrated SPs and examiners’ concern and interest in contributing to helping future generation of doctors, students reflecting on their own clinical skill sets with their individual strengths and weaknesses and how it may impact their future clinical readiness as junior doctors e.g. procedural skills, hands on learning during placements. The examiners compared the intense exam environment to replicating the chaos of clinical work for which student preparation is required. Although all groups focussed on patient safety and readiness for clinical care, their focus was context-dependent where simulated patients drew comparison of medical students with their own doctors, students reflecting on their projected role as doctors and examiners reflecting on their role in student preparation to achieve readiness for practice.

“I feel like when face to face, we are able to see the readiness of the student in terms of their appearances, you see, they will come up with their full overall, doctor coat, with their stethoscope with the equipment that they are supposed to bring. But when it's online, you know, they're just like, doesn't care about how they appear. Therefore, it's just like, more casual. So, we can't really like observe the readiness, like, in a real situation.” (SP-MU1 Female)

“I was really impressed with some of them. I couldn't help once they were out of the room, we had a 10 second interval. So I hope they'll be my doctor one day, they were just so lovely and caring, and being with all everything else that they've got going on that they have to be mindful of time limits and so forth. I was just so impressed with what they could explain to me and it didn't sound rehearsed.” (SP-J Male)

### 5) Implications for future (Ongoing training and assessment for Telehealth)

Examiners and students recognised the strengths and limitations of both OSCE and MONSCE comparing complex consultation to continue in an OSCE format and training for telehealth (online or phone consultations) through online exams, also discussed interest in work-based assessment on the wards.

“But again, I think telehealth is here to stay. So I think that, you know, that online communication skills that our students should be learning, so it’s not unreasonable to assess at least part of their communication online.” (EX-MS Female)

## Discussion

The study aimed to triangulate perspectives from the three key stakeholders involved in and effected by the clinical skills assessment (OSCE or MONSCE) and to consider how it might impact future clinical practice. The themes of *dynamics of discourse*, either in person or online, *perception of realism* through assessments, *preparedness for practice* and *implications for the future*, all highlight the importance of how the perceptions of the examination process is closely linked to the actual outcome the assessments intend to achieve. The theme on *fit for purpose* assessment, and the variability in focus of the three participant groups when considering *of impact on future clinical practice* preparation with ongoing use of Telehealth, highlights how the examination is context dependent for the three participant groups: each group participant reflected on their individual role in the examination process and the whole medicine course.  There was an underpinning focus on patient care and safety.

The study compared the lived experiences of participating in an in-person with online clinical skills assessment. As all groups had participated previously in both OSCE and MONSCE types of assessment, they constantly compared the consequences arising from each of the assessment processes. The participants drew comparison between online and in person assessments with considerations on their advantages such as convenience, no travel required, and suitability for rural and remote set up.  There was less stress for students as they did not face the examiner in the same room or move physical rooms as in an OSCE circuit. They also discussed disadvantages such as difficulty in maintaining student honesty as they had the potential to cheat, less opportunity for face-to-face patient contact, and the inability to have any respectful physical examinations, or to demonstrate empathy during tasks like breaking bad news. The lack of eye contact or inability to recognise body language/ cues was particularly highlighted by all three groups of participants.  This is consistent with previously reported impacts on student-SP discourse, with occasionally difficult transitions to online.^10^^, ^^11^

The *purpose of the assessment* task was also impacted by the online MONSCE such as relatively easier communication during history taking or offering explanations to patients but not possible to demonstrate physical examination skills or in complex clinical counselling such as offering advice to stop drinking alcohol or complex counselling or for breaking bad news. This has also been highlighted in other studies where online OSCE impacted physical examination.^12^ This finding may encourage clinical educators to look for other opportunities such as work-based assessment for clinical examination. Other studies also demonstrated online OSCEs improved history taking and counselling skills, when employed as a formative tool.^13^ All three participant groups - examiners, simulated patients and student groups acknowledged the role of MONSCE when asking patients for history and simple tasks such as offering explanations and consenting for procedures. This was however found to be particularly challenging where astute observations of patient behaviours were required such as body cues, sitting positions, physical appearances or eye contact. Procedural and physical examination skills were not included in the MONSCEs highlighting the need for supplementing these skills with an in-person (perhaps, placement-based) assessment.

The impact of online and examination environment (both physical and virtual) was acknowledged in the context of considering realism of the assessment. SPs compared the OSCE exams to a clinical consultation with a physician and in that context also noted the upcoming role of Telehealth which may continue to be clinically practiced, where feasible. Hence, both SP and examiner groups thought that training for Telehealth is a necessary skill for students (recently recommended by medical regulatory bodies) and MONSCE participation will facilitate that, as it will make students better at assessing and communicating online with patients.^14^ This can impact our current educational and assessment practice to encourage students’ training for providing online consultations in future. When comparing the two exam processes, there was potential noted for continuing the MONSCE which also was the last theme on future directions and applications, even though there was a preference to return to the OSCE model if only one modality was to be employed, also noted elsewhere in literature.^15^^,^^16^

Even though preparation for future clinical practice was not explicitly mentioned, there were repeated references to it in all participant groups (with students voicing concern on their own exam performance so they could transition to the next year level, SPs referring to clinical doctor-patient communication in reference to the online or in-person OSCE and examiners’ motivation to address student preparedness for clinical Tele-Health and in person demonstration of empathy and also being ready for chaos faced in the real clinical world. This emerged as a theme in considering the role of ongoing online and in-person clinical assessments. At the time of writing, many universities that had transitioned to online assessments are considering their ongoing role in continuing them possibly, to supplement the in-person learning and continuing online assessment skills.^17^ There have been other studies suggesting alternatives for OSCE, such as, remote rating by examiners using video-recoding of exam performance,^18^ using web-based interactive, virtual patient simulation with student performance assessed through a computer-generated program^19^ but as far as we are aware not many studies have comprehensively evaluated, with input from simulated patients, examiners and students.

***Limitations of the study and implications for future research***

While the study explores study explores all stakeholder perspectives, it’s limited to evaluation of acceptability and perceived impact of an online assessment. It does not compare and address the validity, reliability or educational impact contributing to the utility of an assessment. Well planned research encompassing available assessments, is required to evaluate overall professional competence, also considering the contribution of formative assessments. This was supported by a systematic review published on online simulation based assessments, highlighting the need for assessment of clinical competence.^20^ Also future research can evaluate the role of online assessments as a formative tool; the online format was employed in this context as a summative assessment, but it may also have a future role to train students for online medical consultations and improve their history taking and clerking skills. Readiness for online consultations is becoming necessary for junior doctors in workforce, and its training should be covered in the medical curriculum. These programs may warrant further evaluation into what skills students acquire and if these skills are retained following graduation.

## Conclusions

Online assessments such as MONSCE, were found to be a useful assessment tool in clinical history talking and consultations requiring patient counselling, not involving complex issues. Physical examination and clinical procedural skills assessment, and, also, complex counselling processes were better suited for in-person examination. Online assessments may have an ongoing role in continuing clinical assessments and may also be useful to promote online consulting skills needed for medical education. There may be a role of assessing clinical engagement of students, observing them in clinical work as a possible means of assessment in future.  Online assessments are an easily auditable, practical and resilient examination.  They not only offer an acceptable and flexible alternative to face-to-face clinical assessment but may also be valuable for assessment of online consultation skills; especially now, as online clinical consultations are becoming part of clinical practice, it is desirable that our future students are trained and have the necessary skills required for online consultants.

### Acknowledgements

We would like to acknowledge and thank the effort of Prof Michelle Leech and A/Prof Julia Harrison for providing feedback on the study and Ms Pauline Forbes for assisting in communication with simulated patients. We also want to acknowledge the students, examiners, and simulated patients for participating in the study.

### Conflict of Interest

The author declares that there is no conflict of interest.

## References

[1] Khan KZ, Ramachandran S, Gaunt K, Pushkar P (2013). The Objective Structured Clinical Examination (OSCE): AMEE Guide No. 81. Part I: an historical and theoretical perspective.. Med Teach.

[2] Harden RM (1988). What is an OSCE?. Med Teach.

[3] Patrício MF, Julião M, Fareleira F, Carneiro AV (2013). Is the OSCE a feasible tool to assess competencies in undergraduate medical education?. Med Teach.

[4] Muntz MD, Franco J, Ferguson CC, Ark TK, Kalet A (2021). Telehealth and medical student education in the time of COVID-19—and beyond.. Acad Med.

[5] Looi JCL, Maguire P, Bonner D, Reay RE, Finlay AJF, Keightley P, Tedeschi M, Wardle C, Kramer D (2021). Conduct and evaluation of final-year medical student summative assessments in Psychiatry and Addiction Medicine during COVID-19: an Australian University Medical School experience.. Australas Psychiatry.

[6] Khalaf K, El-Kishawi M, Moufti MA, Al Kawas S (2020). Introducing a comprehensive high-stake online exam to final-year dental students during the COVID-19 pandemic and evaluation of its effectiveness.. Med Educ Online.

[7] Vygotsky LS. Mind in society: the development of higher psychological processes. In: Cole M, John-Steiner V, Scribner S, Souberman E, editors. Cambridge, MA: Harvard University Press; 1978.

[8] Krueger RA. Focus groups: a practical guide for applied research: Sage Publications; 2014.

[9] Ritchie J SL. Qualitative data analysis for applied policy research. Burgess R.G BA, editor. London; New York: Routledge; 1994.

[10] Chan SCC, Choa G, Kelly J, Maru D, Rashid MA (2023). Implementation of virtual OSCE in health professions education: a systematic review.. Med Educ.

[11] Blythe J, Patel NSA, Spiring W, Easton G, Evans D, Meskevicius-Sadler E, Noshib H, Gordon H (2021). Undertaking a high stakes virtual OSCE (“VOSCE”) during Covid-19.. BMC Med Educ.

[12] Saad SL, Richmond C, Jones K, Schlipalius M, Rienits H, Malau-Aduli BS (2022). Virtual OSCE delivery and quality assurance during a pandemic: implications for the future.. Front Med (Lausanne).

[13] Grover S, Pandya M, Ranasinghe C, Ramji SP, Bola H, Raj S (2022). Assessing the utility of virtual OSCE sessions as an educational tool: a national pilot study.. BMC Med Educ.

[14] Roman P, Ruiz-Gonzalez C, Rodriguez-Arrastia M, Granero-Molina J, Fernández-Sola C, Hernández-Padilla JM (2022). A serious game for online-based objective structured clinical examination in nursing: a qualitative study.. Nurse Educ Today.

[15] Elnaem MH, Akkawi ME, Nazar NIM, Ab Rahman NS, Mohamed MHN (2021). Malaysian pharmacy students’ perspectives on the virtual objective structured clinical examination during the coronavirus disease 2019 pandemic.. J Educ Eval Health Prof.

[16] Lim AS, Lee SWH, Karunaratne N, Caliph S (2020). Pharmacy Students’ perceptions and performance on the use of an online virtual experience tool for practicing Objective Structured Clinical Examinations.. Am J Pharm Educ.

[17] Felthun JZ, Taylor S, Shulruf B, Allen DW (2021). Assessment methods and the validity and reliability of measurement tools in online objective structured clinical examinations: a systematic scoping review.. J Educ Eval Health Prof.

[18] Bußenius L, Harendza S (2023). A simulation-based OSCE with case presentation and remote rating - development of a prototype.. GMS J Med Educ.

[19] Oliven A, Nave R, Gilad D, Barch A (2011). Implementation of a web-based interactive virtual patient case simulation as a training and assessment tool for medical students.. Stud Health Technol Inform.

[20] Ryall T, Judd BK, Gordon CJ (2016). Simulation-based assessments in health professional education: a systematic review.. J Multidiscip Healthc.

